# Predatory Journals and Perished Articles; a Letter to Editor

**Published:** 2017-01-14

**Authors:** Mashallah Narimani, Mehdi Dadkhah

**Affiliations:** 1 Independent Researcher on Library and Information Science, Isfahan, Iran.; 2 Independent Scientist, Information Science and Publication Ethics Scholar, Isfahan, Iran.

**Figure 1:**
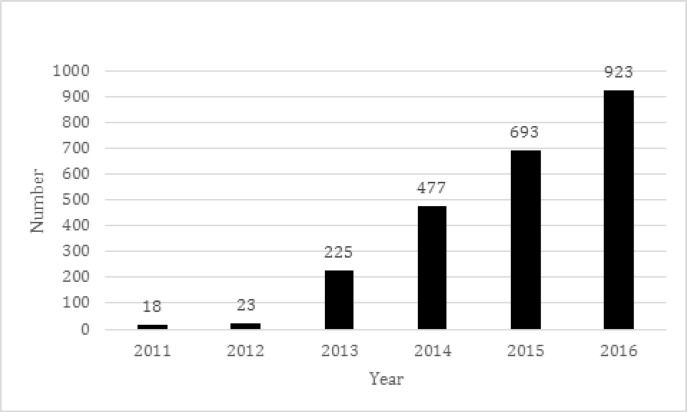
Number of predatory publisher from 2011 until 2016 (4).

In recent years, academic publishing has been faced with many destructive phenomena. “Predatory publishers” (or journals) are one challenge for scholarly publishing. This term was introduced to academic societies for the first time by Jeffrey Beall in 2010 (1). A predatory journal is a journal which publishes papers without peer review, or by an unfair review process and charging publication fees (2). According to Beall’s definition, questionable peer review process and unknown location of the real controlling entity are among the most important criteria for detecting predatory journals (3). In 2016, Beall announced that the number of predatory publisher was growing (4) from 18 in 2011 to 923 in 2016 (figure 1). These statistics convince researchers of the need for more research into dealing with predatory journals. 

Predatory journals have little or no peer review, thus they will be a repository for false principles and bogus research (1). These bogus researches may be dangerous in some domains such as medical sciences, especially emergency medicine. In some countries, the number of published papers are more important than their quality (5). In this situation, predatory authors will publish bogus papers in such journals. Unwary researchers may use published papers in predatory journals for their research and it will lead to establishing their own research based on such previous bogus research. 

By mining the most recent Beall’s list of predatory journals (6), we found that there are 56/1080 (5%) predatory medical journals in his list. It is necessary to state that many fraudulent journals have general domains and publish papers in the medical science domain as well, so we can find many predatory journals and bogus research related to emergency medicine. However, some predatory journals have reputable indexing and their published papers will be indexed in scientific databases. 

Predatory publishers are in the habit of sending huge number of “call for papers” emails to receive papers from authors. In this condition, naive authors will submit their paper to these journals and, as a consequence, predatory journals grow every day. 

**Figure 2:**
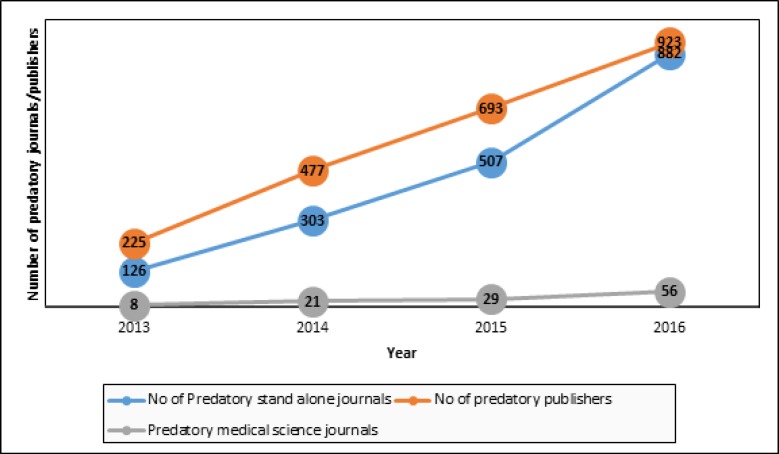
Number of predatory publishers, standalone predatory journals and predatory medical science journals from 2011 until 2016 (7)

It is necessary to establish a suitable educational plan for confronting fraudulent journals. In recent years, the growth rate of such journals has increased, but the number of new registered journals do not appear to have high growth. Figure 2 shows the number of predatory publishers, standalone predatory journals and predatory medical journals (specifically publishing papers about emergency medicine) from 2011 until 2016 (7). According to this figure, we can conclude that predatory journals and publishers have rapid growth.

In conclusion, the best method for dealing with predatory journals is a good educational plan and increasing the awareness of authors about the problem (8). Authors must avoid submitting papers to journals that promise fast publishing by receiving publication charges. Table 1 shows the main criteria for detection of predatory medical journals. To that end, we extracted these main criteria from Beall’s criteria for predatory journal detection (3). 

**Table 1:** Main criteria for detection of predatory medical journals

**No.**	**Criteria**	**Description**
**1**	Contact information	Email addresses of editors are not available and journal uses general email services such as Yahoo or Google.
**2**	Publication charges	Journal uses different types of charges such as publication charges, page charges, author’s charges, fast track charges.
**3**	Similar names	Journal uses a name similar to standout and popular medical journals to confuse authors and cheat them.
**4**	Bogus metric	Journal claims that it has an impact factor, but it is not indexed in Thomson Reuters Web of Science and uses bogus metrics such as Global impact factor (GIF), Scientific indexing service (ISI), etc.
**5**	Call for paper	Journal sends a huge number of spam emails to receive paper from authors. In these call for papers, journal claims that it has a high impact factor and is indexed in valid databases. Some journals have a general name such as “academic research…” and publish papers in medical sciences.
**6**	Review and publishing process	The process of review and publishing is not clear and paying publication charges is more important than quality and publishing standards.
**7**	Number of published papers	The journal publishes many papers in each issue and most of them are outside of journal’s aim and scope.
